# National systemic lupus erythematosus prospective cohort in Saudi Arabia: A study protocol: Erratum

**DOI:** 10.1097/MD.0000000000027039

**Published:** 2021-08-27

*Medicine* is issuing a correction for “National systemic lupus erythematosus prospective cohort in Saudi Arabia: A study protocol”^[1]^ which was published in Volume 100, Issue 30. The article incorrectly appeared as a Research Article: Observational Study article and has since been corrected to appear as a Research Article: Study Protocol Clinical Trial. Authors were contacted and provided an updated SPIRIT checklist. The following additional corrections were also made:

- The abbreviation list initially included the STROBE acronym. The list has been updated to include the SPIRIT acronym.
- A statement in the Methods section initially appeared as “This study design was guided by Strengthen the Reporting of Observational Studies in Epidemiology (STROBE) checklist.[8,9]” The sentence now appears as “This study design was guided by Standard Protocol Items: Recommendations for Interventional Trials (SPIRIT) checklist. [8,9]”
- References 8 and 9 originally appeared as:
  [8] von Elm E, Altman DG, Egger M, et al. The Strengthening the Reporting of Observational Studies in Epidemiology (STROBE) Statement: guidelines for reporting observational studies. Int J Surg 2014;12:1495–9.
  [9] Vandenbroucke JP, von Elm E, Altman DG, et al. Strengthening the Reporting of Observational Studies in Epidemiology (STROBE): explanation and elaboration. Int J Surg 2014;12:1500–24. The references have been updated and now appear as:
  [8] Chan AW, Tetzlaff JM, Altman DG, et al. SPIRIT 2013 statement: defining standard protocol items for clinical trials. Ann Intern Med. 2013;158(3):200-207.
  [9] Chan AW, Tetzlaff JM, Gøtzsche PC, et al. SPIRIT 2013 explanation and elaboration: guidance for protocols of clinical trials. BMJ. 2013;346:e7586. Published 2013 Jan 8.
